# Collecting and Analyzing Patient Experiences of Health Care From Social Media

**DOI:** 10.2196/resprot.3433

**Published:** 2015-07-02

**Authors:** Majid Rastegar-Mojarad, Zhan Ye, Daniel Wall, Narayana Murali, Simon Lin

**Affiliations:** ^1^ Marshfield Clinic Research Foundation Biomedical Informatics Research Center Marshfield, WI United States; ^2^ Marshfield Clinic Nephrology Marshfield, WI United States; ^3^ The Research Institute at Nationwide Children's Hospital Columbus, OH United States

**Keywords:** patient satisfaction, social media, health care, natural language processing, consumer health information

## Abstract

**Background:**

Social Media, such as Yelp, provides rich information of consumer experience. Previous studies suggest that Yelp can serve as a new source to study patient experience. However, the lack of a corpus of patient reviews causes a major bottleneck for applying computational techniques.

**Objective:**

The objective of this study is to create a corpus of patient experience (COPE) and report descriptive statistics to characterize COPE.

**Methods:**

Yelp reviews about health care-related businesses were extracted from the Yelp Academic Dataset. Natural language processing (NLP) tools were used to split reviews into sentences, extract noun phrases and adjectives from each sentence, and generate parse trees and dependency trees for each sentence. Sentiment analysis techniques and Hadoop were used to calculate a sentiment score of each sentence and for parallel processing, respectively.

**Results:**

COPE contains 79,173 sentences from 6914 patient reviews of 985 health care facilities near 30 universities in the United States. We found that patients wrote longer reviews when they rated the facility poorly (1 or 2 stars). We demonstrated that the computed sentiment scores correlated well with consumer-generated ratings. A consumer vocabulary to describe their health care experience was constructed by a statistical analysis of word counts and co-occurrences in COPE.

**Conclusions:**

A corpus called COPE was built as an initial step to utilize social media to understand patient experiences at health care facilities. The corpus is available to download and COPE can be used in future studies to extract knowledge of patients’ experiences from their perspectives. Such information can subsequently inform and provide opportunity to improve the quality of health care.

## Introduction

In the current era of information technology, patients often post their experiences with health care providers to social media websites, similar to reviews of restaurants or hotels. A 2012 survey by the University of Michigan found 65% of the US population was aware of online physician ratings [1]. Another survey by PwC Health Research Institute in 2013 [2] suggested nearly half of all consumers had read health care reviews online and, of those, 68% utilized the information within the review to assist with the selection of their health care provider. The same survey cited 24% of consumers have written a health care review, up from the 7% estimate in a 2011 survey [3].

Besides numerical ratings, the textual content in patient reviews can be a valuable resource for health care providers to improve their services. Data on patient experience is becoming a critical component in the value-based purchasing program proposed by the Center for Medicare and Medicaid Services (CMS) [4]. In contrast to Press Ganey or Hospital Consumer Assessment of Healthcare Providers and Systems (HCAHPS) [5,6], the peer-to-peer nature of patient reviews on social media websites provides a unique perspective for health care providers to understand patient satisfaction. This study is one of a few which focuses on utilizing online peer-to-peer communications to learn about patient experiences and concerns about health care providers [7,8].

Several researchers have studied online patients reviews [7-20], but most of them analyzed doctors rating website [13-20]. Greaves et al [7] conducted a sentiment analysis study on 6412 online comments about hospitals on the English National Health Service (NHS) website in 2010. They applied machine learning approach to classify reviews into positive and negative classes. In addition, Alemi et al [9] studied 995 online comments at the RateMDs website [21] and showed that real-time satisfaction surveys were possible.

Yelp is a popular social media website that allows customers to share their business experiences with other customers. Previous studies suggest that Yelp can be a reliable source to study patient experiences with health care providers [22]. Yelp has made available an Academic Dataset of the 13,490 closest businesses to 30 universities for researchers to explore [23]. Many methodological papers have been published on analyzing restaurants [24-26] using this data set. However, this data set has yet to be studied in the context of health care.

A PubMed search of “Yelp” resulted in only 3 papers. Kadry et al [17] conducted a study to analyze 4999 physicians’ ratings in the 10 most visited websites including Yelp. They found that most patients gave physicians favorable ratings: the average rating was 77 out of 100. Bardach et al [21] found the Yelp ratings correlate well (*P*<.001) with traditional measures of hospital quality (HCAHPS) and suggested that Yelp can be a reliable source to study patient experience. Recently, Butcher [27] reported that health care providers are starting to pay attention to the Yelp ratings. All 3 papers analyzed Yelp ratings but did not utilize the wealth of information contained in the corpus of Yelp reviews.

We addressed this gap by using a corpus of Yelp reviews to characterize patient experience. A "corpus" is a collection of texts presented in electronic form. In this study, we used the Yelp Academic Dataset to construct a corpus of patient experiences. Several natural language processing (NLP) methods and tools were utilized to clean the data and tag the parts-of-speech such as noun phrases and adjectives, and to create parse and dependency trees. A sentiment score for each sentence was also projected and insights from summary statistics of the corpus are presented here.

## Methods

We used 26 health care-related categories (examples include hospitals, urgent care facilities, and medical centers) to extract health care related businesses (a list of categories is provided in Multimedia Appendix 1) from the Yelp Academic Dataset. After identifying 6914 reviews, Stanford Core NLP [28] was used to split reviews into sentences. Porter Stemmer [29] was applied to stem each sentence. Stanford Core NLP was further used to produce parse trees and dependency trees for the sentences and part-of-speech tags for each word. Hadoop was used to run the NLP in parallel to create the corpus. Dragoon Tool was used to extract nouns and adjectival phrases [30]. Sentiment score for each sentence were derived using SentiWordNet [31]. In addition, each sentence was tagged to classify whether or not it was negated. The Hidden Markov Model was used in our negation detection tool [32]. By filtering out terms, which appeared <5 times, 7612 words were selected to form a COPE vocabulary list. The COPE vocabulary list was compared with the consumer health vocabulary (CHV) [33] which is the gold standard in this domain. The CHV covers all health topics. The latest CHV of 2011 contains 158,519 words. To identify co-occurring pairs of terms in each review, we tokenized words and then removed stop words. A Chi-square test was conducted and the odds ratio for each pair for each term which appeared at ≥25 times (empirical cutoff) in the corpus was calculated. Finally, a network of the pairs with high Chi-square (>100), significant *P* values (*P*<.05) and odds ratios >1 was built.

## Results

### Overview

The first observational study of how patients communicate with their peers regarding their health care experiences using the social media website Yelp is presented here. To analyze these communications, a corpus was established and characterized with descriptive statistics.

### Corpus of Patient Experience (COPE)

**Figure 1:**
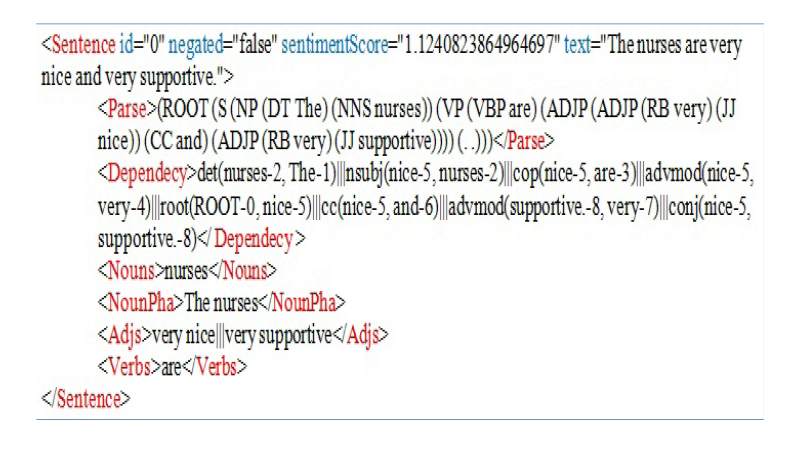
Part-of-speech analysis conducted on each sentence in COPE.

The COPE contains 79,173 sentences from 6914 patient reviews of 985 health care facilities near 30 universities in the United States. The top 10 cities with the most reviews incorporated into COPE are summarized in Table 1. For each sentence in COPE, a part-of-speech analysis was conducted (Figure 1) and made available for future research.

The list of the most commonly encountered nouns, adjectives, and verbs in the corpus and rates of frequency are shown in Table 2.

**Table 1:** The number of health care facilities and reviews from the top 10 cities covered by the COPE.

City	Health care facilities, n	Reviews, n
Palo Alto, CA	123	988
La Jolla, CA	122	872
Pasadena, CA	76	831
Cambridge, MA	50	611
Los Angeles, CA	75	541
Austin, TX	31	239
San Diego, CA	31	252
Houston, TX	58	261
San Luis Obispo, CA	56	235
Seattle, WA	28	255

**Table 2:** The top 20 noun phrases, adjectives, and verbs in COPE (after lemmatization).

Noun phrase	Frequency (per1000 sentences)	Adjectives	Frequency (per 1000 sentences)	Verbs	Frequency (per 1000 sentences)
Time	52.24	Good	52.90	Be	381.42
Doctor	38.20	Great	35.51	Have	197.99
Massage	32.43	Nice	19.89	Go	83.18
Place	31.08	First	17.78	Get	80.35
Staff	30.11	New	16.09	Do	68.72
Office	28.97	Friendly	16.01	Make	40.97
Care	28.68	Few	13.08	See	39.87
Appointment	25.96	Bad	13.02	Take	35.85
Experience	25.45	Sure	11.27	Feel	32.85
Dentist	21.39	Dental	11.24	Give	31.69
Eye	18.79	Little	11.08	Come	31.17
Patient	17.68	Clean	10.90	Say	31.08
Service	16.88	Many	10.82	Tell	30.59
Room	16.50	Professional	10.52	Know	28.49
Insurance	16.47	Last	10.18	Find	23.13
Hour	15.75	Live	9.97	Want	21.78
People	15.66	Medical	9.85	Think	20.36
Surgery	15.19	Next	9.66	Ask	20.25
Pain	14.77	Much	9.25	Recommend	18.99
Review	14.09	Same	8.94	Visit	15.88

### Descriptive Statistics of Reviews in COPE

**Figure 2:**
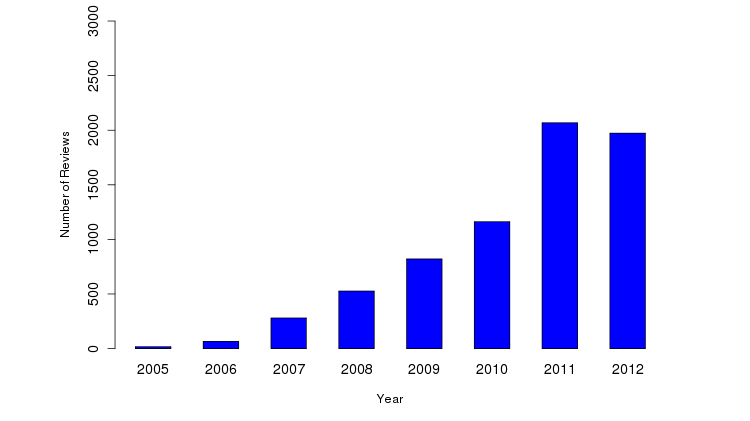
Number of reviews per years.

Over the years, there has been a rapid growth of the number of COPE reviews posted on Yelp (Figure 2). The earliest COPE review was published in 2005, and the most recent was published in 2012. The earlier years, between 2005-2007, were associated with a very high year-over-year growth rate, with a doubling time every 6 months. From 2007-2012, growth stabilized at a rate of 1.5 times annually. Note that 2012 was only a partial year of data collection.

**Figure 3:**
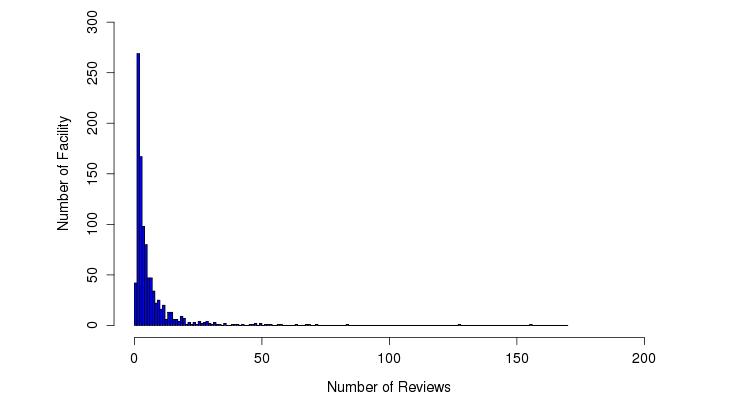
Distribution of reviews.

**Figure 4:**
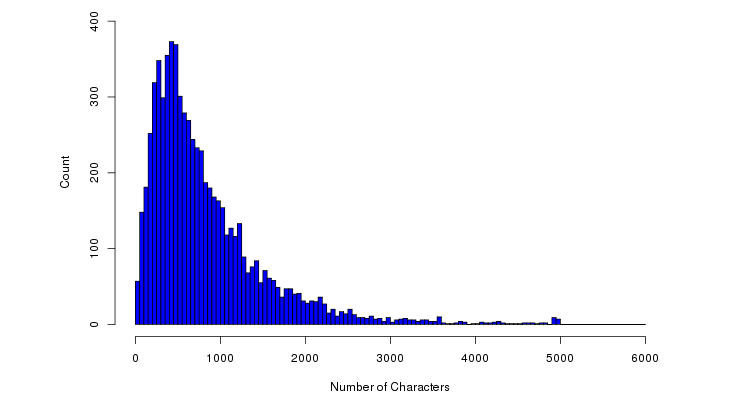
Distribution of review length.

**Figure 5:**
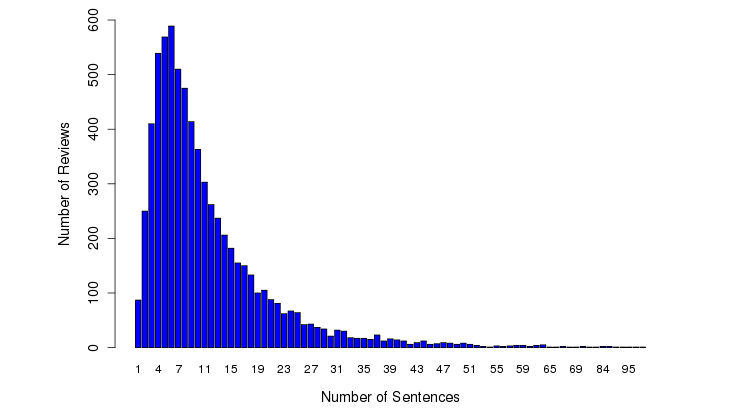
Number of sentences per review.

Although most facilities (93.0%, 916/985) received <20 reviews, 2 facilities (%0.2, 2/985) received >100 reviews (Figure 3). The median length of each review was 635 characters (Figure 4) and the median number of sentences in each review was 9 (Figure 5).

### Consumer Rating and Sentiment Analysis of COPE

**Figure 6:**
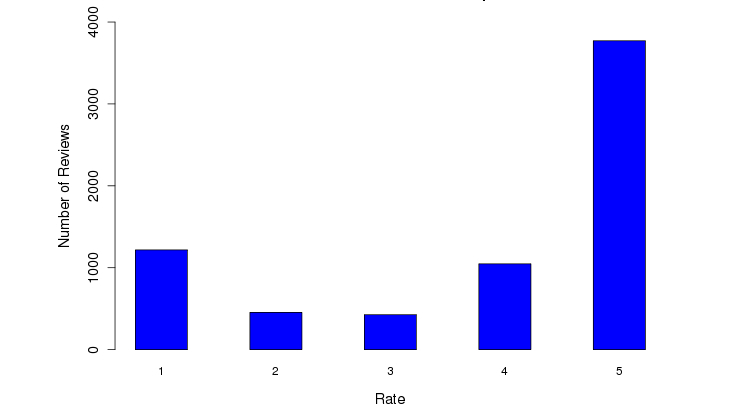
Distribution of the rating scores per review.

**Figure 7:**
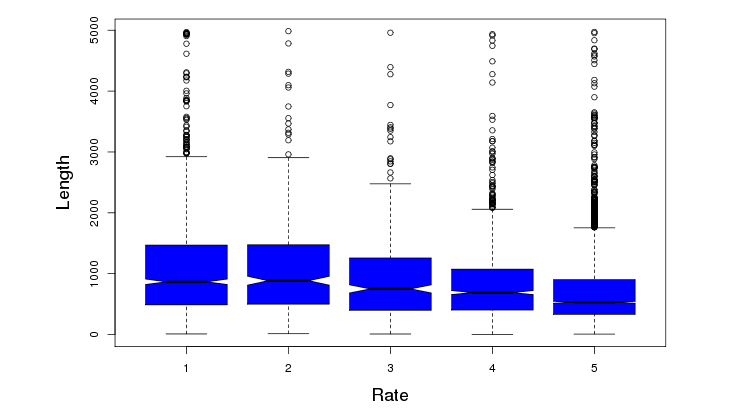
Length of review versus rating score.

**Figure 8:**
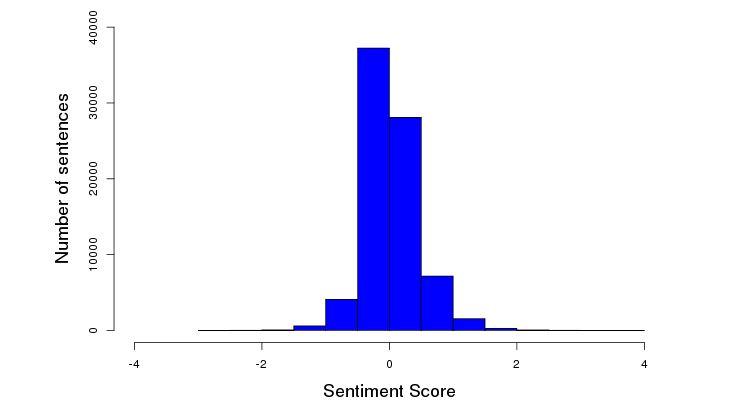
Distribution of the sentiment score per sentence.

**Figure 9:**
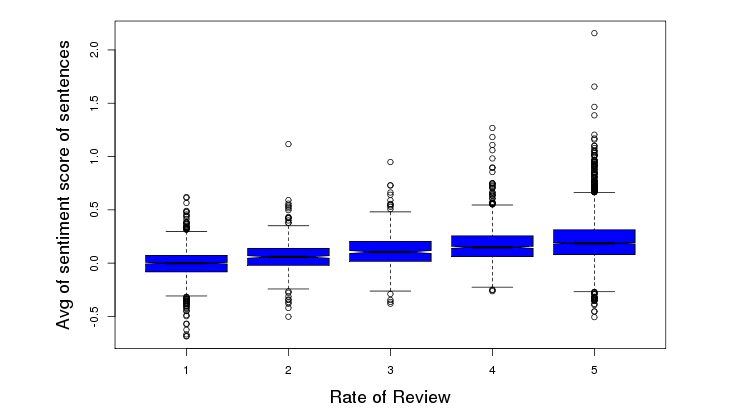
Mean sentiment score of sentences in each review per patient-generated overall rating.

**Figure 10:**
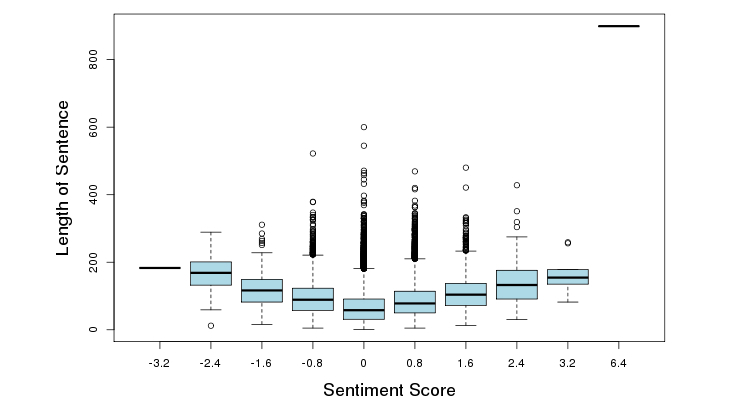
Sentiment score per length of sentence.

On a scale of 1-5 (with 5 being the best), 69.68% (4817/6914) patients rated the facility favorably (≥4 out of 5) (Figure 6). A trend was identified between length of patient reviews and perception of a negative experience (correlation=-.5829, *P*<.001) (Figure 7). Figure 8 illustrates the distribution of sentiment score per sentence. The computed sentiment score was compared with the consumer-generated rating (*P*<.001, Pearson correlation test) (Figure 9). The sentiment score reflects the degree of accumulation of sentimental words in a sentence, which can be signified by *positive* words such as “pleasing” and “perfect,” and *negative* words such as “unhappy” and “disappointing.” Longer sentences tended to carry stronger sentiment score (Figure 10).

### A Consumer Vocabulary Derived From COPE to Describe Their Health Care Experience

**Figure 11:**
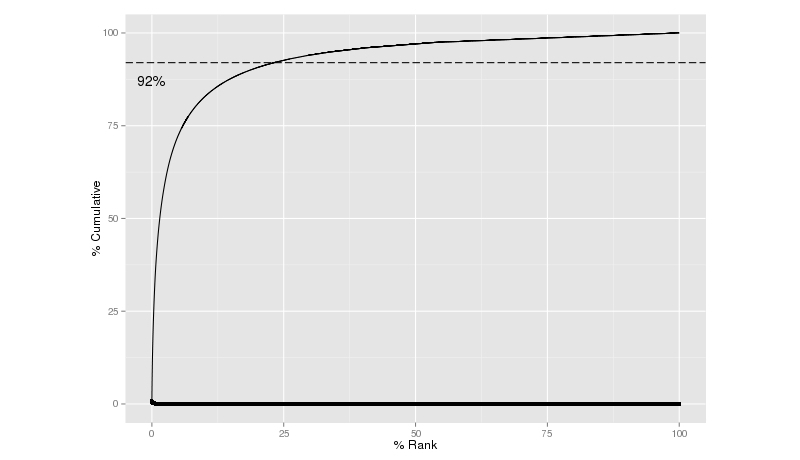
Cumulative usage of terms versus rank of terms.

A total of 25,692 words were derived from COPE. Consistent with vocabulary used in other domains, the top 25% of the vocabulary covered 92% of the usage (Figure 11).

COPE vocabulary was also compared to the CHV [32]. Of all the words in the COPE vocabulary, 8136 (31.67%, 8136/25692) were found in the CHV. The top 20 overlapping and non-overlapping words within the CHV are shown in Table 3.

**Table 3:** The top 20 overlapping and non-overlapping words within the CHV.

Overlapping		Non-overlapping	
Word	Frequency (per 1000 sentences)	Word	Frequency (per 1000 sentences)
Good	53.54	Take	36.43
Time	52.21	Give	31.69
Like	40.91	Tell	30.59
See	39.87	Care	28.68
Doctor	38.20	Know	28.49
Back	35.85	Call	24.70
Great	35.84	Wait	23.98
Feel	35.06	Find	23.13
Massage	32.43	Ask	20.25
Come	31.17	Nice	19.99
Place	31.08	Room	16.50
Work	30.50	Friendly	16.16
Staff	30.11	Visit	15.88
Office	28.97	Help	15.72
Appointment	25.96	Use	14.22
Experience	25.45	Seem	11.74
Look	23.96	Clean	11.55
Dentist	21.39	Check	10.29
Think	20.96	Exam	10.21
Well	19.37	Explain	9.06

A co-occurrence analysis [34] revealed that these words formed a network. For example, the following words formed a tight cluster when patients described their experience with platelet donation (”blood”, “donor”, “platelets”), the snacks offered (“cookie” and “juice”), and the thank-you items given (“movie” and “ticket”) (Figure 11).

**Figure 12:**
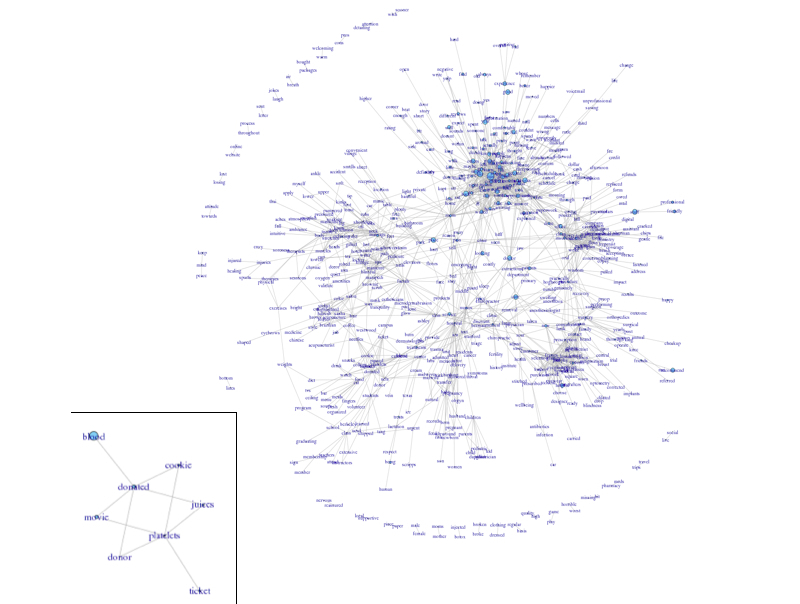
A network of words used by customers to describe their experiences. The size of the node indicates the frequency of the word and the width of the lines indicates the number of co-occurrences of the word-pair in the same review. An example of usage of the word “platelet” is shown in the call-out box.

## Discussion

### Principal Findings

This study yields insightful results following a statistical analysis of 79,173 sentences from 6914 patient reviews of 985 health care facilities. The trend that we observed between length of patient reviews and perception of a negative experience is consistent with a previous study of consumer reviews [35]. Figures 4 and 5 suggest that the texts in COPE are much longer than Twitter (140 characters), which allow more sophisticated content analysis such as identifying the debates among different reviewers in future research studies.

Findings in this study indicate that online reviews could be used to understand important aspects of business from the customers’ point of view. Consistent with a previous report on CHV [33], we also observed that a small vocabulary set (25%) covered a majority (92%) of the content (Figure 10). In examining Table 2 and considering the most frequent noun phrases (ie, time, doctor, massage, place, staff, office, care, appointment) we can see important aspects of health care business as the most frequent terms used by patients. Table 3 further suggests that the COPE vocabulary list covers more about the patient experience with health care providers, including sentiment words such as “nice” and “friendly” and experiential words such as “wait” and “visit”. Moreover, the co-occurrence analysis revealed a statistical “wordnet”, which can recover some interesting associations in the context of health care (Figure 11).

Our comparison of the computed sentiment score with consumer-generated rating (Figure 9) showed good correlation between the mean sentiment score of sentences and patient-generated. This result further validated our computational approach for sentiment analysis and the consistency of rating by the patients.

### Limitations

The data source of the Yelp Academic Dataset used herein was associated with the following study limitations. First, it was geographically biased with businesses surrounding 30 universities in the United States. Table 1 suggests that the data set is highly concentrated in the east and west coasts, and Texas. Second, the date range of the reviews was limited from 2005-2012. There were no updates available from the Yelp Academic Dataset. However, this dataset is the accessible Yelp data for academic research, since the Terms of Service by Yelp Inc prevents any automatic data retrieval of Yelp contents. In addition, there is an implicit selection bias toward “patients” (we cannot verify they are truly patients) who choose to write a review at Yelp. Moreover, the credibility and content of some reviews has been challenged by physicians and provider organizations on whether the review content truly reflects an unbiased patient experience or is representative of the actual quality of care [27].

### Conclusions

The created and characterized COPE corpus includes patient reviews, ratings, parse trees, dependency trees, and a vocabulary list. The COPE corpus further enables future policy studies, such as using machine learning techniques such as unsupervised learning of topic analysis or supervised analysis of classifications [7] to analyze the patient reviews in the context of six domains of quality established by the Institute of Medicine [36]. COPE is available for academic use [37].

## Appendix

Multimedia Appendix 1 List of the 26 Yelp health care categories in the study.

## Abbreviations

CHV: Consumer health vocabulary
COPE: Corpus of Patient Experience
HCAHPS: Hospital Consumer Assessment of Healthcare Providers and Systems
NLP: Natural language processing
