# Supplementary material for: Collecting and Analyzing Patient Experiences of Health Care From Social Media
Source: JMIR Res Protoc. 2015 Jul 2;4(3):e78. doi: 10.2196/resprot.3433 (PMC4526973; doi:10.2196/resprot.3433)
Supplement: Multimedia Appendix 1 [file resprot_v4i3e78_app1.pdf]

Hospitals

Urgent Care

Health and Medical

Medical Centers

Obstetricians and Gynecologists

Sports Medicine

Orthopedists

Doctors

Family Practice

Allergists

Internal Medicine

Pediatricians

Laser Eye Surgery/Lasik

General Dentistry

Chiropractors

Physical Therapy

Eyewear and Opticians

Optometrists

Ophthalmologists

Periodontists

Dentists

Oral Surgeons

Massage

Massage Therapy

Naturopathic/Holistic

Acupuncture
